# The Cardiac Rehabilitation Psychodynamic Group Intervention (CR-PGI): An Explorative Study

**DOI:** 10.3389/fpsyg.2018.00976

**Published:** 2018-06-20

**Authors:** Claudia Venuleo, Gianna Mangeli, Piergiorgio Mossi, Antonio F. Amico, Mauro Cozzolino, Alessandro Distante, Gianfranco Ignone, Giulia Savarese, Sergio Salvatore

**Affiliations:** ^1^Department of History, Society and Human Studies, University of Salento, Lecce, Italy; ^2^Istituto Scientifico Biomedico Euro Mediterraneo, Mesagne, Italy; ^3^“San Giuseppe di Copertino” Hospital, Azienda Sanitaria Locale Lecce, Copertino, Italy; ^4^Department of Human, Philosophical and Educational Science, University of Salerno, Fisciano, Italy; ^5^Department of Cardiology, “Antonio Perrino” Hospital, Azienda Sanitaria Locale di Brindisi, Brindisi, Italy; ^6^Department of Medicine, Surgery and Dentistry “Scuola Medica Salernitana”, University of Salerno, Fisciano, Italy

**Keywords:** myocardial infarction patients, post-infarction condition, affective meanings, psychodynamic group intervention, verbatim transcripts

## Abstract

**Objective:** An explorative study focusing on the process of a Cardiac Rehabilitation Psychodynamic Group Intervention (CR-PGI) addressed to myocardial infarction (MI) patients is discussed. The study aimed at analyzing whether the treatment based on CR-PGI serves as a communicational context within which MI patients are enabled to explore new interpretations of their post-infarction condition.

**Methods:** The intervention, divided into 12 weekly one-hour group sessions, was addressed to MI patients recruited within a Public Hospital of southern Italy. Each session was audio-recorded and lexical correspondence analysis (LCA) was applied to the verbatim transcripts, in order to provide a map of the evolution of the communication exchange occurring over the 12 sessions.

**Results:** The findings showed that the discourses associated to the first eight sessions differed from the discourses of the last four sessions. Two main transitions occurred. The first concerns the response to the infarction, first interpreted as a process of affective elaboration and afterwards as practical management of the functional aspects associated with the condition of MI patients. The second concerns the nature of the change and contrasts a lifestyle-oriented model with a social role approach, which refers to social, legal, and medical practices related to the acknowledgment of being an MI patient.

**Conclusion:** The findings offer preliminary support to the capacity of CR-PGI to work as a context where new meanings for the biographical rupture of the MI can be explored. Consistently with the rationale of the model, the intervention seems to have promoted the emergence of new ways of feeling and understanding one’s condition.

## Introduction

Cardiovascular diseases are the main cause of death both in the United States, with 600 cases per 100,000 people each year (Jiaquan et al., 2016) and in Europe, affecting three times more men than women (Canto et al., 2012). According to the findings of the Framingham Heart Study, among myocardial infarction (MI) patients, one out of four presents a relapse in the following 10 years (Berger et al., 1992), with negative prognostic effects; a large percentage of those who survive an acute event become chronically ill, needing a serious rehabilitation process. Studies suggest that MI is combined with a significant reduction in health-related quality of life (HRQL) (Schweikert et al., 2009). Secondary prevention after MI is crucial in reducing risk and suffering. Recently, the INTERHEART study of nearly 30,000 individuals from 52 countries revealed that modifiable risk factors – dyslipidemia, smoking, hypertension, diabetes, alcohol consumption, obesity, stress, and diet – are the strongest predictors of heart disease as manifested by MI (Yusuf et al., 2004). These modifiable risk factors also affect the probability of further MI episodes and mortality (Esposito et al., 2003).

In recent years, HRQL is increasingly accepted by health organizations and physicians as a complementary measure of the medical effectiveness of interventions (Bullinger, 2002; Schweikert et al., 2009). Cardiac rehabilitation (CR) programs have become an integral part of the standard of care in modern cardiology. Their scope has shifted over the last four decades from the emphasis on exercise therapy to comprehensive secondary prevention strategies managing risk factors. This involves physical activity, tobacco cessation, nutritional counseling, aggressive coronary risk-factor management and therapeutic education to life changes (Mampuya, 2012).

However, it is increasingly recognized that non-compliance to prescribed medication is common in patients with heart failure (Cline et al., 1999; Ye et al., 2012) and, more in general, in clinical practice (Nikolaus et al., 1996; Venuleo and Guacci, 2014). Despite the awareness of the need for a change in life style, patients are often not able to change. A sizeable proportion of the population are aware of the dangers of a high fat diet without apparently being either willing or able to do much about it (Johnston, 1999); smokers are aware of the harmful consequences of smoking on their cardiac condition and health in general, yet are unable to break the habit (Hirani and Newman, 2005).

Studies on chronic illness emphasized how the experience of a disease or chronic illness affects not only the health condition of the patients, their habits and lifestyle but, more broadly, the way of making sense of the experience (Bonomi, 2003), and of relating to the world in the process of adapting to their new situation. The person’s biological impairment is often seen in terms of rupture, which creates discontinuity in personal experience (Boeijea et al., 2002; De Luca Picione et al., 2016). The psychodynamic theory highlights how people tend to live and react to disruptive events (like a critical state of health like MI) in terms of emotional, affect-laden interpretations, rather than in a merely rational way (De Luca Picione et al., 2016; Salvatore and Venuleo, 2017): events, persons, acts, discourses, and any other element of life – including heart attack and the change it imposes – acquire existential meaning in the light of the subjective affective frame by which people interpret their being in the world (Salvatore and Venuleo, 2008; Salvatore and Freda, 2011; Venuleo et al., 2016, 2017). Affective meanings are encapsulated with respect to the cognitive processing of the information concerning the changed situation; consequently, they are unable to learn from experience. And this is just a different way of saying what psychoanalysis has claimed about the unconscious, namely that it does not contemplate time, negation, and novelty (Freud, 1899/1955; Matte Blanco, 1975). At the phenomenological level, the inertial valence of affects is there for all to see, and with it, people’s tendency to interpret the new in terms of the past (Salvatore and Venuleo, 2017).

Such characteristics of affective meaning-making can offer an interpretative key to understand people’s difficulty in coping with the change required by the post-heart attack condition – as well as any other kind of major transition. Indeed, when affective meaning-making is dominant – as in the condition of deep rupture – the person tends to be emotionally blind to the new elements occurring at the level of their social and personal role. They recognize the new aspects at a verbal level, but this awareness is unable to become body and action, namely to be assimilated deeply by the person – it works as if it were the representation of something else, concerning others. This means that the interpretation of the new reality – the one imposed by the heart attack rupture – tends to be carried out within the affective meanings comprising the before-rupture subjective frame (Salvatore and Venuleo, 2017). And this frame may not enable the patients to manage the new condition and to carry out the important lifestyle changes needed for the sake of risk reduction.

These considerations lead to the recognition of the role that the psychodynamic-oriented intervention can play in supporting MI patients during the post-infarction stage. Psychodynamic-oriented intervention is recognized as elective treatment for helping people to elaborate the affective implications triggered by and associated with critical events, of both a physical and psycho-social nature (Ventegodt et al., 2007). The central idea is that an increased awareness of the role played by affect in patients’ meaning-making and therefore in shaping their response to pain might facilitate the process of elaboration of such affects and therefore the adaptation to the new condition and challenges of life (Adams et al., 2006).

Psychodynamic-oriented interventions are widely used in Western societies and are not limited to the psychotherapy domain. Their adoption and utility have been documented with regard to patients expressing different types of pain (Turk et al., 2008): e.g., cancer (Straker, 1998; Ludwig et al., 2014), multiple sclerosis (Muston, 2017), diabetes (Winkley et al., 2006), and fibromyalgia syndrome (Scheidt et al., 2013). However, to the best of our knowledge, the few studies that have focused on the use of psychodynamic-oriented interventions in the context of CR are aimed at addressing specific risk factors rather than allowing a more general elaboration of the meaning by which the patients interpret the biography rupture. For instance, Michal et al. (2013) describe the protocol of an intervention aimed at evaluating the effectiveness of a psychodynamic care approach – the Psychodynamic Motivation and Training (PMT) program – particularly in improving the physical activity of MI patients. The study by Albus et al. (2011) aimed to evaluate the effectiveness of a Stepwise Psychotherapy Intervention with patients with cardiovascular disease, using supportive individual psychotherapy and combined psychodynamic and cognitive–behavioral group therapy in reducing symptoms of depression and Type D pattern (negative affectivity and social inhibition). According to the authors, the aim of the psychodynamic technique is “to systematically deal with individual maladaptive cycles of interaction as well as to elaborate alternative ways of action by newly gained understanding of one’s emotional experiences in the context of the group process itself” (p. 217). However, the study does not enable us to understand how the intervention works.

Thus, it is relevant and useful to promote studies aimed at developing and validating rehabilitative interventions based on the psychodynamic rationale and meant to help patients subject to infarction to elaborate the affective meaning associated with their new medical condition, and the biographic rupture that the latter produces in their life.

Accordingly, the present study is aimed at presenting a brief psychodynamic-oriented group intervention – the Cardiac Rehabilitation Psychodynamic Group Intervention (CR-PGI) – designed to be performed in a hospital context, the latter assumed as one of the elective contexts of the CR. The paper is divided into two parts. First, the rationale of the intervention is presented; then an explorative study focused on the treatment process is discussed, in order to describe the modality through which the intervention works, and in so doing, providing preliminary evidence of its construct validity.

### Rationale

The CR-PG intervention is conceived as an interpersonal environment (i.e., a group) in which the affect-laden meaning triggered by the biographical rupture of the MI can be enacted and thereby elaborated. This is expected to promote the emergence of new ways of feeling and understanding one’s condition (Carli, 1987; Salvatore, 2011), and therefore to enable lifestyle changes that are consistent with the patient’s new medical state.

#### Peculiarities of Affective Meaning-Making

As has been said above, the rupture due to MI experience prompts an affect-laden form of meaning-making, namely a lived interpretation of the critical event, and more in general of the person’s relationship with the world (Salvatore and Venuleo, 2017). Thus, one can see patients that feel and think of themselves, and also behave, in terms of impotence – i.e., as if the MI event had made them completely devoid of resources with no chance of defending and improving their health and making their life better; in other cases, patients seem to simply deny the critical medical condition they have to cope with, seeing themselves in terms of omnipotence – i.e., as if nothing actually bad could ever really happen to them (and consequently they will tend to underestimate the risk factors associated with their lifestyle). Again, in other cases the patient might experience the critical event in terms of guilt/punishment – i.e., “I deserve what happened” – or in terms of appearance – “people will see me so weak and useless now” – and so forth. These affect-laden meanings are more than emotional reactions – they are *modes of interpreting one’s life condition* (Salvatore and Venuleo, 2008); it is for this reason that we refer to them in terms of *affective meaning-making* (Salvatore and Freda, 2011).

There are three main features of affective meaning-making distinguishing it from the cognitive, rational-based way of thinking (Salvatore and Venuleo, 2010). First, affective meaning-making is inherently *holistic* – that is, it concerns the entire relation between the subject and the world as a whole (e.g., the sense that “nothing changes in my life” does not refer to a discrete object of experience, but connotes the entire field of experience as a single totality). Second, affective meaning-making works in *hypergeneralized* and *homogenizing* ways – it assimilates the facets of experience according to basic, embodied identity-laden and emotionally laden classes of meanings (positive-negative; powerful-powerless) that provide the subject with the real sense of reciprocal engagement between the self and the world. This means that the affective meaning is a way of giving with world an existential value for the subject itself (Salvatore, 2015). Third, as a result of the points above, the affective meaning is *non-semantic*, which means it tends to be only weakly constrained by semantic (and functional) norms: instead, it tries to overcome the differentiated aspects of reality (e.g., the different incidence of risk associated with smoking and not smoking) – in the philosopher’s words, the affective meaning is the night in which all cows are black (Tonti and Salvatore, 2015). More specifically, affective meaning-making leads experience to be interpreted by making pertinent the facets of the reality (and only those) that – according to the affective meaning involved – are relevant for the subject (e.g., a person that sees himself as powerless will feel and shape beliefs about the world as an ongoing sequence of threatening, powerful elements too big and strong to deal with). This leads to the last point – affective meaning-making cannot *be taught by experience*, i.e., it is blind to the informational content of the response provided by the world (other people, environment, shared knowledge). This is because, as we said, on the one hand it does not recognize the semantic content and on the other hand, it concerns how one would wish to be in the world, rather than how the world actually is.

The characteristics of affective meaning-making outlined above lead us to recognize that, when this kind of psychological dynamics is at stake – as in the case of the rupture due to a critical state of health like MI – the cognitive elaboration of the new condition of illness is not enough. It is unable, in itself, to reach and affect the embodied, identity-laden and affect-laden level of meaning-making that channels the patient’s sense of self and therefore the course of his/her action.

#### How Affective Meaning-Making Changes

Affective meaning-making cannot be taught by experience, but it can learn from experience. According to the psychodynamic theory, affective meaning-making changes as a result of the exercise of the reflective function (Taylor and White, 2000; Jurist, 2005). Psychoanalysis has extensively discussed this cognitive process and a review of that literature is outside the scope of our paper. Rather, consistently with the purpose of the current paper, we will merely observe that the reflective function can be viewed as comprising two complementary processes. On the one hand, the current subject’s affective embodied mental state is associated with a semantic representation (e.g., “I feel happy” as the representation of the current embodied state of happiness) (to move from the thing-representation to the word-representation, to use the Freudian terminology). This function of representability of the mental state is consistent with the mentalization function (Bateman and Fonagy, 2012), the capacity of perceiving oneself and others as characterized by mental states (i.e., assumption, beliefs, intentions, hopes). Through this notion, the psychodynamic standpoint places great emphasis on the representability of the affects (more than beliefs, reasoning and perspective), which create a relationship (Jurist, 2005) and recognizes the here and now of the intersubjective transactions taking place in the clinical intervention as the main instrument of change (Karterud, 2015). As Stern (2004) suggested, it is the experience as the originally lived (the present moment) that provides the raw material for a possible later verbal accounting, thus for an explicit, declarative, knowledge that can be represented and elaborated. The representational content linked to the affective meaning can then be elaborated cognitively, in accordance with the axiological and functional norms provided by the cultural milieu. On the other hand, this process of semantic elaboration produces a new pattern of meaning (e.g., “I am happy, and I am right to be so, given I am a survivor; but this should not have hampered me in seeing the negative facets of my condition”), which, given its linkage with the triggering of affective meaning, gives rise to its modulation.

#### How CR-PGI Works

Cardiac Rehabilitation Psychodynamic Group Intervention does not adopt a normative approach. In other words, it does not pursue the promotion of a pre-defined set of meanings and beliefs as to the post-infarction situation. By contrast, it is aimed at promoting the patient’s autonomous capacity of rethinking and managing his/her condition, supporting the capacity of improving the quality and efficacy of his/her self-management. The CR-PGI pursues this non-normative approach through three main functions.

First, by favoring the expression of the person’s current affective embodied mental state, so that an embodied aspect can be linked to a symbolic representation, and can thus be gradually elaborated, rather than merely enacted. Since we can only be aware of the here and now (the present moment of experience), the expression concerns the present moment of the person’s affective state at any given point in a session.

Consider the following excerpt of a session:

Instructor: “What are your expectations about this group?”
Participant A: “I think you are going to help us to face our condition”
Instructor: “What kind of help do you expect?”
Participant A: “The heart attack disrupts your life. So how do I begin…”
Participant B: “Let’s start with the medical recommendations. We have to stop smoking, to eat healthy…”
Participant C: “I have to reduce my weight… I like to eat”
Other remarks about lifestyle changes follow
Instructor: “So you are worried about lifestyle changes imposed by your state of health. What kind of constraints are you facing?”
Participants E: “I have no clear what I can do and what not. Surgeons are so quick when you go for a check-up. It seems it is never the right moment to talk about your concerns.”
Participant B: “And it is not easy to share your feeling in the family”
Instructor: “Do you want to explain why?”
Participant B: “It is difficult. Well…. they are already concerned about your health and if I talk them about my concerns all I achieve is that they look more at me: what I eat, how much I work, ….”
Participant D: “I’m experiencing the same situation at work. My colleagues say: “Are sure you want to go back to work….but I feel so useless…”
Participant B: “It makes me feel like a child. (They say) you shouldn’t do this and that….I know it’s for my health but I feel so angry sometimes.”
Instructor: “If I understand rightly, what are you saying is that the change to manage is also related to what happens around you, in terms of relationships because this make you feel bad (angry, useless).”
Participants begin to speak about their own feeling of being overlooked or over-controlled

In the example, the instructor presses for the participants’ different feeling and expectations to emerge (“*What kind of help do you expect?*”; “*What kind of constraints are you facing*?”) and makes them explicit (“*So you are worried about lifestyle changes imposed by your state of health*”; “*If I understand rightly, what are you saying is that the change to manage is also related to what happens around you, in terms of relationships because this make you feel bad*”).

Second, the instructor tries to facilitate the acknowledgment of the affect related to one’s illness and the interpretation of the related meaning, so that participants can become aware of how this meaning affects the ways they feel and act. The interpretations are grounded on three main sources: the contents of the discourse (what the participants choose to talk about), the free associations (participants are invited to relate whatever comes into their minds during the session – viewed as the expression of the subjective experience of the present moment), and the relational model acted out in the here and now of the group session (the ways the participants relate to the instructor, to the other participants, to the group setting).

A participant (A) arrives late at the group session. He seems very irritated and he starts to describe the different circumstances that justify his being late.
Instructor: “You seem very agitated, as if you expect to be reproached by us.”
Participant A: “It is true…I feel as if I’ve made an unforgivable mistake. It is kind of like when doctors and family talk about the lifestyle change they expect from me.”
Instructor: “How does that make you feel?”
Participant A: “I feel really despondent…”
Instructor: “Are there other situations where you’ve felt like that?”
Participant A: “I felt like that when I was late for my daughter’s birthday….; and also when I broke my leg and I left my wife alone to look after our sons and their busy schedules. I felt very bad that I was unable to take care of my loved ones…”
Instructor: “It seems to me that in all the situations you are talking about, a feeling of guilt is involved… you feel you’ve been bad with the components of this group and with your loved one but you are also saying that the first person who suffer the consequences is yourself”
Participant A: “Yes, I am angry at myself about all the things I’ve missed.”
Participant B: “I am very impressed with what is being said now. I am coming to realize that sometimes I stick with the expectations of others, also about my health, because they force me to be aware of what I risk losing. It is important for us to be precise, to eat healthy food, to stop smoking.”
Instructor (addressed to all the participants): “What do you think about this?”

In the excerpt, the instructor encourages a participant to share the subjective experience of being late for the session and the related feeling (“*How does that make you feel?*”), explores other situations the patient associates to that feeling in order to understand what kind of meaning orients the interpretation of the experience in the different circumstances (“*Are there other situations where you’ve felt like that?*”), interprets the affective meaning underlying the patient’s discourses (“*It seems to me that in all the situations you are talking about, a feeling of guilt is at stake…*”) and encourages its elaboration (“*you are also saying that the first person who suffers the consequences is yourself*”), also soliciting the other participants to give an opinion (“*What do you think about this?*”). In this way, patients can critically review their way of coping with difficult situations (“I am angry at myself about the things I’ve missed” and “It is important for us to be precise, to eat healthy food, to stop smoking”).

Third, the instructor supports the acquisition of habits and strategies more consistent with the requirements of reality related to the medical condition (*normative function*). To this end, the instructor:

1. makes explicit reference to the medical recommendations (i.e., “smoking, having a high fat diet and being sedentary increases your risk of heart disease”);
2. favors social support among the participants: they are encouraged to share tangible resources, give advice, answer questions that they can use to cope with the daily problems related to their medical condition (i.e., how to go about getting the invalid benefit);
3. gives and encourages empathic support, including listening, encouragement, reassuring, and comforting for illness-related concerns, to permit the expression of emotions and worries that patient feel it is difficult to discuss with their family and friends and to reduce feelings of personal inadequacy.

Throughout the encounters, the three main functions of CR-PGI are contemplated and both interpretative and supportive interventions are made by the instructor in relation to the specific and contingent dynamic expressed by the participants. It is worth noting that, due to the non-normative character of CR-PGI, there are no specific, predefined questions that the instructor addresses to the participants, since the reflexive function of CR-PGI concerns the analysis of the meanings through which the participants intimate they understand and manage their experience. The instructor takes on a “not knowing” position (Anderson and Goolishian, 1992; Bateman and Fonagy, 2006), like that of a therapist who lets the clients express themselves in the way they choose, rather than replacing it with the therapist’s own assumptions or notions of the reality that should be described (White, 2007). So, a way for the instructor to start the meeting may be: “What are your expectations about this encounter?” or “what do you think it is important we talk about?” which allows the different points of view of the participants to emerge.

#### The Setting of the Intervention

The CR-PGI intervention is organized in a semi-open group setting for two main reasons. First, it is widely recognized that a group setting with people experiencing the same stress or sharing some fundamental therapeutic qualities, such as opportunities for disclosure, empathic connection, shared goals, and psychological adjustments (i.e., decrease of distress and sense of social isolation) to life challenges (Davison et al., 2000). Investigations of the effects of support group participation showed that heart victims taking part in support groups display more health benefits than non-participating controls or controls on a waiting list (Dracup, 1985). Second, a group approach may be more cost-effective than individual intervention and more consistent with the hospital’s need to provide support to all the patients in care.

The intervention is organized in 12 one-hour weekly sessions, with an overall length of 3 months. The group is expected to be conducted by a clinical psychologist, due to the specificity of the competences required to perform the psychological functions of the CR-PGI (comprising both supportive and interpretative interventions) and the complexity of the emotional process that we can expect to characterize the members. Work of this kind requires the instructor to be familiar with the transference process, to be able to deal with countertransference, to cope with intimacy and to represent and solicit the reflexive-analytic attitude in the group (Foulkes, 1984; Karterud and Bateman, 2012). Furthermore, the instructor has to be able to apply specific functions related to the management of group dynamics, which are rooted in group-analysis and psychodynamic group therapy (Anthony and Foulkes, 1965; Yalom, 1995; Karterud and Bateman, 2012): e.g., the instructor sets the process norms, encourages turn taking and group interaction, and fosters the interpersonal group process as a vehicle for change.

### The Case Study

#### Aims and Hypothesis

The CR-PGI is based on three main tenets: (a) the CR-PGI promotes a form of communicational exchange among the participating outpatients that prompts a change in how group members interpret their post-infarction condition; (b) such a change modulates the underlying affective meaning grounding the whole sense of the existential and identity rupture conveyed by the heart attack; (c) the changes mentioned in the previous points go beyond the group setting, enabling outpatients to improve their quality of life in terms of health.

The study focuses on the rationale’s first tenet: it is designed to analyze whether the treatment based on CR-PGI serves as a communicational context within which post-infarction patients are able to explore new interpretations of their state. To this end, the case study provides a map of the evolution of the communication exchange occurring over the 12 sessions of a rehabilitative group with post-infarction outpatients, based on the CR-PGI. This is done in order to check the following two hypotheses, drawn from the CR-PGI rationale:

First, we expect to find an evolution through time of the meaning that the group associates with their post-infarction state.

Second, we expect this evolution to be consistent with the general function of the rehabilitative intervention, namely with promoting a healthier lifestyle and realistic forms of adjustment to the new medical condition.

As one can see, these two hypotheses are explorative – they do not consist of precise expectations as to the specific content of the new pattern of meaning, according to the non normative CR-PGI approach to change. Rather, it is just expected that a change will occur (HP1) and that this change has a general valence of consistency with the promotion of healthy conditions and realistic adjustment to the new medical condition (HP2).

Finally, it is worth recognizing that the focus of the study concerns just one (the first) of the CR-PGI tenets, namely a necessary but not sufficient condition of the method’s construct validity. Therefore, the present study has to be considered just a preliminary step in the direction of CR-PGI validation.

#### Framework: Structural Analysis of the Group Exchange

In order to see if and what evolution of meaning occurred over time in the CR-PGI case a two-step strategy of analysis was implemented.

First, the analysis carried out *a structural map of the meaning variability* characterizing the whole CR-PGI communication exchange. As intended here, meaning variability is the set of different themes (i.e., beliefs; opinions; combined statements; verbal images) referring to the subject that is the focus of the communicational exchange (in this study, the post-infarction condition). As indicated above, the analysis was designed to map the meaning variability *structurally*. To this end, the analysis focused on the main *dimensions of semantic variability* underpinning the set of contents that were active in the group exchange. Each dimension of semantic variability can be conceived of as a component/quality of the subject which was made pertinent by the participants in the communication exchange and provides space for a plurality of statements and positions. For instance, the component COLOR is a quality of a dress and the source of the potential plurality of statements like “it is gray,” “it is green,” and so forth. To give a more relevant example, insofar as the group makes pertinent the impact of the heart attack on a person’s life, then this semantic component provides space to express different statements/connotations on this aspect (e.g., one participant might say “life changed totally” whereas another might say “after the first little while everything returned to normal”). Thus, the structural map of the meaning variability goes beyond the descriptive level of the content analysis and identifies the semantic structure generating the variability of the contents (Visetti and Cadiot, 2002; Salvatore et al., 2017b).

Second, it was esteemed whether the semantic variability is associated with time, namely with the course of the communicational exchange performed by the group session by session. It is worth highlighting that in so doing the hypotheses were checked at a structural rather than a descriptive level. In other words, the study did not analyze whether a change in the content of the communicational exchange occurred between different moments of time. Rather, the study tried to identify a “deeper,” latent level of meaning change, consisting of a variation of the pertinent semantic components underpinning the communication exchange. This methodology was adopted because whereas the change at the level of contents is contingent to the local circumstances of the communicational change (e.g., it can emerge as a result of the mere fact that the group leader focused the group discussion on that theme), the main components of semantic variability are quite generic structures. Each of them underpins a plurality of themes at the same time (e.g., the semantic component “life change due to the infarction” traverses and shapes a plurality of themes such as one’s image, the future prospects, what happens on the job, the family, the reaction of others, and so on). As a result, the structural level of the components of semantic variability is less affected by contingent aspects; one could therefore interpret their evolution over time as a marker of the actual trajectory of the meaning-making promoted by the CR-PGI group.

Finally, it is worth pointing out that we consider the instructor’s utterances an active component of the semantic variability which characterizes the communication exchange. For these reasons, the structural analysis was applied to the whole CR-PGI communicational exchange, and not limited to the discourses of each single participant. This choice is consistent with the intersubjective nature that we recognize in the sensemaking process (Salvatore and Venuleo, 2013; Salvatore et al., 2017b) and, more widely, with the psychodynamic perspectives which, with different concepts and language, share the emphasis on the intrinsically intersubjective nature of the clinical process (Mitchell, 2014; Orange et al., 2015) and the view of group matrix as a narrative shared by all members (Foulkes, 1971; Karterud and Bateman, 2012).

## Materials and Methods

### Participants

Participants were recruited among the outpatients of the cardiac division of a public hospital located in Southern Italy. The age range for the selection (from 18 to 70) was established to cover potentially all the outpatients from young to middle age, consistent with the hospital’s need to provide support to all the patients in care. This choice was also consistent with the psychodynamic scholars who recognize that both supportive and exploratory interventions are suitable for adults ranging from young to old (Garner, 2002).

To be eligible for the study, patients had to meet the following criteria: (a) have suffered from MI or heart failure (within the last 6 months); (b) show no major medical issues (e.g., malignancy, neurological disorders) or psychological problems (e.g., psychotic spectrum disorders, mental disability); (c) show no features preventing them from participating continuously in group sessions (i.e. constraints due to inability to reach the site).

Participants (*N* = 7; 2 women) recruited were aged between 48 and 67 (mean age = 58.9; SD = 6.6). Their educational levels ranged from primary school (two participants), to middle school (two participants) to high school (three participants); two individuals were employed; the others were jobless or retired. The group started with six patients. After the first session, two patients dropped out. A new member was recruited and involved in the sixth session. During the intervention, the number of participants hovered between 3 and 6.

### Procedure

The recruitment took 9 months (from March to November 2015). Patients sent to the group by the hospital cardiac ward were among outpatients released in the last 3 months after the acute stage of treatment. Consistently with the semi-open nature of the group, new patients were accepted once the group started, however, only in the case of substitution of patients who had abandoned the group and, however, only during the first half of the treatment (i.e. the first six sessions).

Patients released by the cardiac ward were contacted by phone in order to invite them to an individual interview at the hospital. During the interview, each patient was informed about the general purpose of the intervention and the voluntary nature of participation. No incentive was given. During the interview, patients agreed to participate and signed the informed consent to the audio recording of the encounters for research purpose.

The intervention lasted 12 sessions (1 session each week) over 3 months.

This study was approved by the Extraordinary Commissioner of the Local Health Agency (ASL) in Lecce (Italy).

### Data Analysis

Each session was audio-recorded. The textual corpus obtained from the verbatim transcript of the communicational exchange performed over the 12 sessions was used as dataset. This includes the utterances of the instructor and of the two members who dropped out, consistently with the idea that each participant plays an active role in the strengthening (or weakening) of the latent meanings informing the discursive exchange.

The data set underwent a computer-aided quanti-qualitative strategy of analysis aimed at mapping the semantic structure underpinning the communicational exchange occurring throughout the 12 group sessions. This strategy views meaning as consisting of sign transitions, namely associations between significances over time – i.e., the meaning is not held in sign A, but by what other signs sign A is associated with (i.e., what signs are followed by and follow sign A) in the concrete contexts of speech (Salvatore et al., 2017a,b). In the case of texts, the sign transition assumes the forms of syntagmatic associations, namely co-occurrences between words within the same contextual units (e.g., a paragraph of the text).

The co-occurrence between words is carried out by means of a multidimensional procedure of analysis [lexical correspondence analysis (LCA)] applied to the whole corpus under investigation and irrespective of the discourse of each participant. It should be noticed that LCA does not apply to the single lexical forms in the text but to the lemmas obtained by a lemmatization procedure. Lemmas are the labels indicating the lexical forms/classes a word refers to, regardless of its syntactic form (Pottier, 1974). For instance, the lexical forms “go,” “goes,” “went” will be transformed, as in a common dictionary, in the single lemma: “to go.” The lemmatisation therefore helps to greatly reduce the lexical variability of the text, thus allowing a more functional application of the procedures of analysis. The multidimensional procedure is applied to the data matrix composed of the segments into which the text is divided (i.e., paragraphs) as rows, lemmas as columns and presence/absence values in cells. The procedure is implemented by means of T-LAB software^1^. The version used in this analysis was 16-Plus.

Broadly speaking, LCA breaks down and reorganizes the relations between lemma in terms of a multidimensional structure of opposed factorial polarities; where each polarity is characterized by a set of lemmas that tend to co-occur and do not occur in the event of the occurrence of an opposite set. Accordingly, this structure can be interpreted as the operationalization of the semantic structure of the textual corpus under investigation, with any factorial dimension to be seen as a marker of a dimension of semantic variability. The interpretation of the factorial dimensions is carried out in terms of the global meaning envisaged by the set of co-occurring lemmas associated with each polarity.

In order to analyze the communication over the session’s temporal span with regard to the semantic structure, the textual corpus was divided into three *temporal blocks*: Block 1 = sessions 1–4; Block 2 = sessions 5–8; Block 3 = sessions from 9 to 12. The three blocks were established to conventionally distinguish the beginning, the middle and the last part of the intervention. Consistently with the purpose of the study, we wondered whether changes occur in how group members interpret their post-infarction condition as a result of the CR-PGI process, but no specific hypotheses were made on the specificity of the semantic structures characterizing the three phases. The temporal block was used as an illustrative variable, namely a variable that is introduced after the LCA extraction of factorial dimension, in order to provide an external source of description of the latter. LCA provides an estimation of the level of association between each factorial dimension and each temporal block (parameter used: *V*-test, interpretable as a *z*-score).

## Results

### The Dimensions of Semantic Variability

The main output of the LCA is outlined below. More specifically, for the two main factorial dimensions, and for each of their polarities, the lemmas with the highest level of association (*V*-Test) are reported (cf. **Tables 1**, **2**) as well as their interpretation in terms of dimension of semantic variability. Lemmas (in italics in the text) are translated from Italian (the language spoken by the group members). The dimensions of semantic variability are labeled in Roman capitals; the polarities in majestic font.

**Table 1:** Lexical correspondence analysis (LCA) output.

Emotional elaboration		Practical management	
Test value^a^	Lemmas	Test value^∗^	Lemmas
-8.5631	*Aspect*	13.5129	*Disability*
-7.0317	*Point*	13.1674	*Check*
-6.5864	*Issue*	13.0358	*Income*
-5.8480	*To live*	11.8818	*Inps*
-5.7924	*Anger*	11.1126	*Board*
-5.7704	*To manage*	11.0421	*Asl*
-5.4860	*Sensation*	9.7707	*Ordinary*
-5.4522	*Series*	9.5230	*To recognize*
-5.4346	*Life*	9.3618	*To center*
-5.1896	*Event*	9.0234	*To belong*
-5.1085	*To happen*	8.3386	*Basis*
-4.9660	*Feeling*	8.3266	*Demand*
-4.9076	*Complex*	8.3180	*Patronage*
-4.8643	*To understand*	8.2771	*To say*
-4.7436	*To wait*	8.2186	*Certified*
-4.7066	*Change*	7.9261	*Month*
-4.5788	*To found*	6.8113	*Visit*
-4.5467	*Situation*	6.6752	*To send*
-4.4990	*Tool*	6.5400	*To pour*
-4.4928	*Stuff*	5.9467	*Doctor*
-4.3287	*Unique*	5.7856	*Year*
-4.3183	*To take*	5.2783	*High*

*Characterization of the first factorial dimension. ^∗^Highest levels of association standard scores (V-Test)*.

**Table 2:** Lexical correspondence analysis output.

Lifestyle		Social status	
Value test^∗^	Lemmas	Value test^a^	Lemmas
-10.5781	*To cook*	12.2679	*Check*
-8.4832	*Kitchen*	12.2271	*Disability*
-8.4519	*Wife*	11.4473	*Income*
-8.1686	*To eat*	10.8653	*To center*
-7.7608	*House*	10.7339	*To recognize*
-6.1425	*Minute*	9.3768	*Inps*
-5.4104	*To die*	9.3616	*Asl*
-5.2751	*Pasta*	8.6342	*Ordinary*
-5.2638	*Week*	8.1909	*Board*
-5.2411	*Sister*	7.7438	*To belong*
-5.1095	*Pleasure*	7.5722	*Matter*
-4.9160	*Morning*	7.2125	*Certified*
-4.9157	*Son*	6.8662	*Disability*
-4.7977	*Day*	6.4794	*Patronage*
-4.7800	*Evening*	6.3462	*Demand*
-4.5371	*To stay*	6.0905	*To pour*
-4.4783	*Fish*	5.8264	*Stuff*
-4.3363	*To concentrate*	5.7315	*Basis*
-4.2888	*To happen*	5.4901	*Series*
-4.0178	*Mother*	5.2991	*Question*
-4.0164	*To devote*	5.1904	*Aspect*
-4.0065	*Husband*	5.0792	*To wait*
-3.9852	*Heart attack*	5.0594	*Different*
-3.9849	*To raise*	4.7701	*Point*
-3.9659	*Night*	4.6662	*Today*

*Characterization of the second factorial dimension. ^∗^Highest levels of association standard scores (V-Test)*.

#### First Dimension. Reaction to the Infarction: Emotional Elaboration Versus Practical Management

The first dimension concerns the reaction to the heart attack. Two basic opposing responses to the illness mark the polarities of the continuum, showing the semantic variability that this dimension underpins – on the one hand, the response of *emotional elaboration*; on the other hand, the response of *practical management* of the consequence of the critical event (**Table 1**).

##### Emotional elaboration

The associated lemmas seem to refer to the handling and elaboration (*to manage, to react, to support, to wait, to accept, to reassure)* of feeling and emotion (s*ensation, feeling, anger, anguish, emotive, simply, complex, difficult, unpleasant, strong*) related to the changes (*change, situation, behavior, context, system*) produced/required by the heart attack. Clinical intervention appears as a useful means to this goal (*group, goal, to reflect, to understand*).

Examples of discourses organized by the emotional elaboration side of this semantic component are: “I feel anger with myself because, for too long, I ignored my body’s wake-up call”; “It is very unpleasant to feel you are overlooked as a child”; “I feel that these group sessions are helping me to manage my anguish aroused by the change I have to face.”

##### Practical management

Lemmas seem to refer to the practical management of the functional aspects associated with the condition of MI patients, both at the medical level (*hospital, visit, doctor, cardiologist, chief, pathology, to cure, to operate*), the economic level (*to spend, income*), and on a civil-legal level (*demand, certificate, disability, check, pension, license, INPS-National Institute of Social Previdence, ASL-Local Health Agency*).

Examples of discourses are: “I am trying to get the disability benefit”; “you have to go to the other office if you want to obtain a quicker answer”; “I went to the doctor last week and she told me my blood levels are better now.”

#### Second Dimension. Nature of the Change: Lifestyle versus Social Status

The second dimension concerns the content of the change produced by the infarction. Here the opposition is, on the one hand, the view of the change as concerning habits and lifestyle, versus, on the other hand, the change as a matter of social status (**Table 2**).

##### Lifestyle

The lemmas associated with this polarity (e.g., *to cook, cooking, to buy, to prepare, to eat, pasta, fish*) seem to entail the view of the infarction as an event triggering a disruptive and constraining modification of habits and consolidated way of living (in particular, in the food area), charged with high existential costs (*to be dead, heart attack, to operate, to recover*). Other lemmas (*wife, sister, son, home)* refer to the family context in which the efforts to change lifestyle take place.

Examples of discourses are: “Before the heart attack, I smoked a lot of cigarettes to manage my stress at work; now I am trying to reduce my work and to enjoy my time off”; “I have to reduce my weight, but it is very difficult if the refrigerator in your home is full of cold cuts and cheeses”; “My wife bought a heart smart cookbook.”

##### Social status

Lemmas associated with this polarity refer to social, legal, and medical practices related to the acknowledgment of being an MI patient (*to recognize*, *to manage, to determine, to ask, to interest, check, invalidity, income, pension, INPS-National Institute of Social Security, ASL-Local Health Agency*). The illness is recognized as a condition which poses limits and reduces one’s capacity to respond to the requirements of one’s role, thus as having an impact on one’s social identity.

Examples of discourses are: “I can’t go back to my job... so, finally, I went ahead and just asked for invalid status”; “My pension is not enough to cover the needs of my family”; “It is very difficult to accept being recognized as an invalid.”

### Semantic Component and Time

**FIGURE 1:**
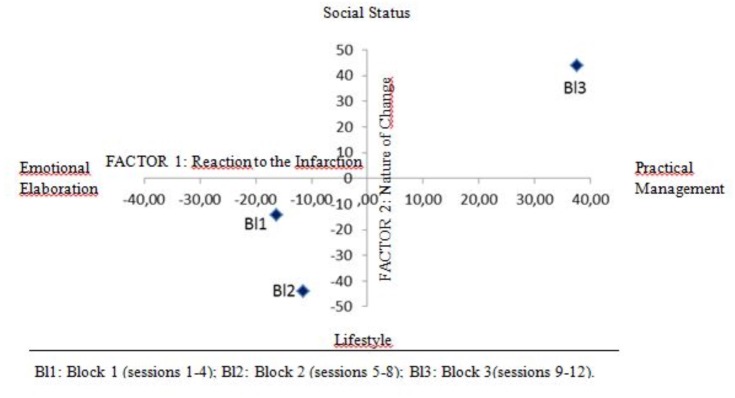
Position of the temporal blocks along the two factorial dimensions.

The three temporal blocks (i.e., sessions 1–4, 5–8, and 9–12; cf. Data Analysis) show a different association with both factorial dimensions obtained by the LCA (**Table 3** and **Figure 1**). More particularly, both the first and the second temporal blocks (Bl1 and Bl2) are significantly associated with the negative polarity (Emotional Elaboration) of the first factorial dimension (-16.26 and -11.55, respectively, *z*-score) as well as the negative polarity (Lifestyle*)* of the second factorial dimension (-14.16 and -44.12, respectively). Inversely, the third period of time (Bl3) is associated with the opposing polarities – Practical Management and Social Status – of both factorial dimensions (first dimension: 37.57; second dimension: 43.95).

**Table 3:** Association factorial dimensions × Temporal blocks.

	Test value^∗^	
Temporal blocks	Factorial dimension 1	Factorial Dimension 2
	*Reaction to the infarction*	*Nature of the change*
Bl1 (sessions 1–4)	-16.26	-14.16
Bl2 (sessions 4–8)	-11.55	-44.12
Bl3 (sessions 9–12)	37.57	43.95

*^∗^Z-score*.

## Discussion

The structural analysis of the verbatim transcripts based on the LCA showed two main dimensions of semantic variability underpinning the communicational exchange among group participants in the CR-PGI intervention. One dimension consists of the opposition between two ways of responding to the event of the infarction (Emotional Elaboration versus Practical Management). The other semantic component can be interpreted as concerning the change induced by the heart attack (Lifestyle versus Social Status)

In short, one can see that the meaning-making produced by the group exchange seems to be characterized by the pertinentization of two extremely basic issues: where the rupture is (inside/outside) and what it consists of (medical issue versus identity issue).

The analysis of the association between these two basic dimensions of semantic variability and the temporal blocks is consistent with both the study’s explorative hypotheses. As regards the first hypothesis, the group’s communicational exchange showed a major change over time, more particularly between the first two temporal blocks (i.e., the first eight sessions) and the last one. The change concerns both dimensions of semantic variability. In the first eight sessions, the group’s meaning-making is focused on the emotional experience of the heart attack and on its negative, constraining consequence on one’s habits and lifestyle; in the last sessions (9–12), the group exchange shifts: on the one hand, the focus becomes the management of the practical (medical, economic, legal) issues induced/associated with the new socio-sanitary state; on the other hand, the infarction is treated as an event with implications for the participants’ identity.

As regards the second hypothesis, one can see that the trajectory of the group meaning-making mapped by the analysis is consistent with the expectation that the change of meaning promoted by the group serves the rehabilitative aims of the intervention, namely to promote a healthier lifestyle. The group spends the first two out of the three temporal blocks talking about the depressing disruptive rupture produced by the heart attack – in its emotional meaning as well as in the compelling and constraining negative consequences on one’s lifestyle. Then, the group shifts to a different area of meaning, the post-infarction state as a new identity status, namely a new way of being-in-the-world (Salvatore and Venuleo, 2017) which involves the social and public sphere and requires new operative and functional tasks of adjustment. To use an image, the group’ s meaning-making seems to have drawn a trajectory moving from the focus on loss – the life before that will never come back – toward opening one’s gaze to the future – the new adjustment challenge that one has to address.

Before concluding, three specifications are worth adding here. First, the recognition of the shift between the semantic components does not allow us to conclude that there is a functional linkage between the two areas of meaning (response to the infarction and nature of the change). Thus, one can only state that the results are consistent with the CR-PGI rationale, according to which it is possible to represent one’s emotions symbolically and bring this representation into the communicational exchange to enable affective and cognitive change. According to this framework, one can conjecture that it is *thanks to the fact* that the group has promoted the representability of the affective meanings of loss associated with the MI event that patients were enabled to re-interpret it in terms of adjustment challenge.

Second, it has to be underlined that this is not a specific clear-cut behavioral outcome (like the decision to stop smoking), but it is an area of meaning that can be viewed as functional to the aim of promoting some form of realistic commitment to adjusting to the new medical condition. Indeed, a preliminary condition for committing to healthy lifestyles is that the patient is able to think of his/her own present condition in terms of the future.

Finally, it has to be highlighted that the trajectory of meaning-making outlined above cannot be generalized – the specific way the CR-PGI group under analysis worked has to be considered. A different CR-PGI group might show a different trajectory of meaning-making. What can be generalized is the more abstract pattern of meaning-making that this case can be seen to exemplify, namely the fact that the CR-PGI intervention is able to promote a form of change in the meaning associated with the experience of MI and that such a change supports the patient’s commitment to face up to the new social and medical condition (for the view of the generalization of idiographic cases in terms of abstraction, see Salvatore and Valsiner, 2010).

## Conclusion

The aim of this study was twofold. First, to present the rationale of a psychodynamic intervention (CR-PGI) designed to favor the awareness and elaboration of the affective meaning through which MI patients interpret and manage their state of health. The second aim was to outline an instance of the communicational exchange through which intervention works. To this end, an explorative study concerning a group of patients was presented. The findings of the study were consistent with the hypothesis supporting CR-PGI construct validity – namely the basic idea that a CR intervention that takes into account the affective meaning associated with the medical condition helps patients to develop more competent and realistic attitudes toward their condition.

From this standpoint, our study represents a contribution to the line of thought that underlines the role of affective meaning in the way patients represent and manage their state of illness, a role that has so far been rather marginal in the field of rehabilitative interventions. As to the clinical implications, the study suggests the usefulness of integrating psychological strategies of interventions focused exclusively on risk reduction with psychological strategies aimed at helping patients to recognize and elaborate the emotional impact of the medical event.

### Limitations

Needless to say, this study is only a preliminary step in the validation of CR-PGI. As with all qualitative research, its conclusions cannot be generalized, since the results depend on the contingency of the intersubjective meaning-making process activated within the group, made up of specific participants, within a specific hospital. It is plausible that the participants’ age range affected in some ways the emergence of the specific topics that characterize our group. For instance, it was observed that the older the patients, the more likely it is that discourses focus on the experience of multiple losses simultaneously, i.e., not only the loss of health or disability, but also adjusting to retirement, reduced income, grandparenthood, second careers, loss of spouse or friends (Santrock, 2002; Myers and Harper, 2004). On the other hand, the age of members may not be the only source of the variability of the meaning-making process that can characterize a CR-PGI group and other sources of meaning variability should be considered (e.g., the level of trust toward the hospital, the levels of social support perceived by the members outside the group, the personal style of the instructor). As observed above, the findings can be generalized on theoretical grounds, via abstraction. Further studies are required both for a deeper understanding of how CR-PGI intervention can work, how it can be affected by contextual (organizational characteristics, setting parameters), psychosocial (group climate), and psychological factors (e.g., personality traits) as well as what outcome it is able to produce.

## Ethics Statement

This study was carried out in accordance with the recommendations of the Extraordinary Commissioner of the Local Health Agency (ASL) of Lecce (Italy): Number 202, February 19, 2015 with written informed consent from all subjects. All subjects gave written informed consent in accordance with the Declaration of Helsinki. The protocol was approved by the Extraordinary Commissioner of the Local Health Agency (ASL) of Lecce.

## Author Contributions

CV contributed to the conception and design of the study, interpreted the data, drafted the manuscript in consultation with SS. GM developed the main research question, recruited the patients who participated in the study, and collected the data. PM analyzed the data and reviewed and revised the work for intellectual content. AA, AD, and GI provided feedback regarding the study design. MC and GS provided feedback regarding the choice of analysis. AA, AD, GI, MC, and GS provided feedback regarding ethical considerations and critically reviewed the manuscript prior to being submitted to this journal. SS was the primary supervisor of the project, contributed to the conception and design of the study, and reviewed the manuscript. All authors approved the final version of the manuscript to be published and agreed to be accountable for all aspects of this work.

## Conflict of Interest Statement

The authors declare that the research was conducted in the absence of any commercial or financial relationships that could be construed as a potential conflict of interest.

## Footnotes

1 www.t-lab.com
